# Preservation of Traumatic Completely Amputated or Avulsed Body Parts in the First Aid Setting: A Scoping Review

**DOI:** 10.7759/cureus.81998

**Published:** 2025-04-10

**Authors:** Eunice Singletary, Jorien Laermans, Jen Heng Pek, Pascal Cassan, Daniel Meyran, David Berry, Kaushila Thilakasiri, Therese Djarv

**Affiliations:** 1 Emergency Medicine, University of Virginia, Charlottesville, USA; 2 Department of Public Health and Primary Care, Leuven Institute for Healthcare Policy, Mechelen, BEL; 3 Emergency Medicine, Sengkang General Hospital, Singapore, SGP; 4 IFRC Global Reference First Aid Reference Center, French Red Cross, Paris, FRA; 5 Department of Kinesiology, Saginaw Valley State University, Saginaw, USA; 6 Ministry of Health, Ministry of Health Holdings, Colombo, LKA; 7 Emergency Medicine, Karolinska Institute, Stockholm, SWE

**Keywords:** amputation, avulsion, preservation, replantation, scoping review

## Abstract

Traumatic amputations or avulsions are physically and emotionally devastating events that may result in long-term disability. Despite increasing global incidence, optimal prehospital preservation strategies are underutilized, reducing the likelihood of successful replantation. This scoping review was performed as part of the International Liaison Committee on Resuscitation's (ILCOR) continuous evidence evaluation process to update the 2025 ILCOR Consensus on Science with Treatment Recommendations. Our aim was to scope the research literature on prehospital preservation methods used for amputated or avulsed body parts and their impact on replantation success. We searched PubMed, Embase, CENTRAL, CINAHL, ClinicalTrials.gov, and the International Clinical Trials Registry Platform from inception through February 26, 2025, for studies comparing approaches to preserve completely amputated or avulsed body parts in the out-of-hospital setting. Data on first aid intervention characteristics, ischemic times, and replantation outcomes were summarized. We concurrently reviewed first aid guidelines and participant manuals from global organizations to assess strategies taught for the management and storage of amputated body parts. Thirty-nine publications were included in the final analysis, with the majority (73%) being case reports. No randomized controlled trials were identified that directly compared different cold storage techniques (e.g., ice-water immersion vs. dry cold storage). Key findings emphasized the importance of cooling amputated parts after wrapping with saline-moistened gauze and double-bagging to extend ischemic tolerance. This review identified an extensive evidence base suggesting that nonfreezing cold preservation of amputated or avulsed body parts as soon as possible after injury is associated with survival of replantation and improved function and cosmesis. Training first aid providers, standardizing prehospital protocols, and increasing public awareness could enhance patient outcomes and expand access to replantation globally. However, additional studies are needed to identify alternative means of sustained cold storage of amputated body parts, especially in low-resource first aid settings.

## Introduction and background

The complete amputation or avulsion of external body parts (e.g., digit, hand, and arm) or soft tissue is a physically and emotionally traumatic experience that can lead to long-term disability and disfigurement. Between 1990 and 2019, the global incidence of traumatic amputations increased from 11.37 million to 13.23 million [1]. The most frequent age group for traumatic amputations is zero to five years, followed by 51-55 years [2]. Fingers are most frequently amputated (91%), followed by toes (5%). Most injuries occurred in the home (56%) due to doors, bench or table saws, and power lawnmowers. In a complete amputation or avulsion of a body part or tissue, the part is entirely severed from the body, leaving no connecting tissues like muscles, tendons, or skin. By contrast, a partial amputation or avulsion has residual connections such as muscles, blood vessels and skin, which enhance the likelihood of surgical repair and functional recovery. Replantation is the surgical reattachment of a body part that has been completely severed from the body and has become more widely available in many countries. Although there are many indications and contraindications for replantation, the first aid management of the amputated part is key to the decision. The initial first aid is directed toward the care of the injured person, including management of life-threatening bleeding and shock. Care of the amputated or avulsed body part may not be considered, and in some cases, the amputated part may be lost or not transported with the patient, leading to prolonged tissue ischemia times.

Several factors beyond the initial prehospital care have been shown to influence the replantation decision [3-7]. Crush and avulsion injuries often result in extensive devitalized soft tissue, making replantation less likely to succeed. Distal amputations have higher success rates compared to proximal amputations involving larger muscle masses, partly due to the complexity of reconnecting multiple structures. Older patients typically have comorbidities such as cardiovascular disease and diabetes that impair healing and increase the risk of postoperative complications. Smoking is a poor prognostic factor in replantation due to its negative effect on vascular health and healing. Other considerations include the potential for the replanted part to regain functional utility, the patient’s occupation and personal needs, and the availability of a surgical team with microsurgery experience.

The ischemia time, or the duration between the amputation and the restoration of blood flow, is critical to the replantation decision [3,7]. Amputated tissues containing skeletal muscle will survive for approximately six hours without perfusion, while digits, which lack skeletal muscle, can tolerate ischemia for 12 or more hours before replantation [3]. However, the preservation of an ischemic amputated limb or digit is considered by replantation surgeons to be an important factor that can improve the potential for successful replantation surgery and can extend the tolerable period of ischemia between injury and replantation [4-7]. Preservation is recommended by cooling the part without freezing and transporting it with the patient to a center capable of performing replantation surgery. When replantation is not an option, skin from the preserved amputated or avulsed body part can sometimes be used as a biological dressing or as a tissue source for grafting and reconstruction [8, 9]. The orthopedic replantation literature describes recommendations for “properly” preserving an amputated body part by wrapping the part in saline-moistened gauze, placing it in a plastic bag or airtight container, and putting the bag or container in a second bag or container of ice and water [7]. However, it has been reported that only 35% of patients with complete traumatic amputations present to an emergency department with “properly preserved” amputated body parts, making it difficult for surgeons to offer replantation when it would otherwise be an option [10,11]. When amputated parts are not preserved, the viability of the part decreases with time, making replantation either not feasible or with residual loss of function. This can severely impact a person’s ability to perform daily activities, maintain employment, and create psychological distress and societal/familial challenges.

Given the lack of a comprehensive review of how best to manage and preserve a completely amputated or avulsed body part, this topic was prioritized by the First Aid Task Force of the International Liaison Committee on Resuscitation (ILCOR) for a scoping review. The Population-Intervention-Comparator-Outcome (PICO) question for this review is as follows: “For adults and children with a traumatic complete amputation or complete avulsion of a body part (e.g., digit, hand, extremity) or soft tissue in the out-of-hospital setting (P), does any approach to the preservation of the amputated or avulsed body part for possible replantation or attachment (I) compared with another approach (C) change clinical outcomes including attempted and successful replantation (O)?” We sought to identify the type and volume of research that has already been undertaken, what factors related to prehospital preservation of completely amputated or avulsed body parts are being reported, and what outcomes or gaps in research are identified.

## Review

Methodology

Study Design

ILCOR uses a continuous process to evaluate evidence and develop treatment recommendations for resuscitation and relevant first aid topics. This scoping review was conducted in accordance with ILCOR methodological standards [12] and is reported in accordance with the Preferred Reporting Items for Systematic Reviews and Meta-Analysis for Scoping Reviews (PRISMA-ScR) checklist [13]. The completed PRISMA-ScR checklist is available in Appendix A. The scoping review complied with a prespecified plan created by members of the First Aid Task Force of the International Liaison Committee on Resuscitation (Appendix B).

Eligibility Criteria

All identified records were screened against the inclusion and exclusion criteria, as shown in Table 1.

**Table 1:** Inclusion and exclusion criteria PICOST: Population, Intervention, Control, Outcomes, Study design, and Timeframe

PICOST item	Inclusion	Exclusion
Population	Adults and children with complete amputations (fingers, toes, digits, hand, foot, arm, leg); adults and children with complete avulsions of soft tissue; animal studies	Infants and neonates; manikin studies; partial amputations or avulsions; degloving injuries; surgical amputations; crush injuries (as these typically result in surgical amputation); mangled extremities; internal avulsions (i.e., tendons, nerves)
Intervention/exposure	Any type of preservation of the amputated/avulsed body part, e.g., rinsing or cleaning; dressing (dry or moistened) to protect the body part; cooling the tissue (cryotherapy)	Delayed preservation (e.g. perfusion, artificial blood); surgical specific preservation (e.g. free flaps)
Comparator	Any other type of tissue preservation	
Outcome	Any clinical outcome, including, but not limited to, successful replantation, successful use of avulsed tissue for covering the defect	
Study designs	Randomized controlled trials (RCT)s; non-RCTs Interrupted time series; controlled before-and-after studies; cohort studies; case report or series; animal studies; conference abstracts; trial protocols; systematic reviews; scoping reviews	Narrative reviews (no systematic searching of the literature)
Timeframe	All years/language if there was an English abstract	

Information Sources and Search

Search strings were developed in collaboration with an information specialist at the Belgian Red Cross’ Centre for Evidence-based Practice (JL). The strings comprising both index terms and text words can be found in Appendix C. The following databases were searched for relevant studies: PubMed, EMBASE, Cochrane CENTRAL, Cumulative Index of Nursing and Allied Health Literature (CINAHL), ClinicalTrials.gov, and the World Health Organization International Clinical Trials Registry Platform (WHO ICTRP). Databases were searched from their inception date until February 26, 2025. In addition, reference lists of all included records were scanned for additional relevant records.

Because we anticipated an abundance of peer-reviewed literature on the replantation of amputated body parts and factors contributing to its success, our gray literature search focused on the identification of current first aid guidelines from ILCOR member organizations and other known first aid organizations.

Selection Process

Each title and abstract, and subsequently each full text, were independently double-screened using Covidence software [14]. JL screened each of the 10,157 records, whereas EMS, JHP, PC, DCB, and KT each screened one-fifth of the records. Disagreements were resolved by discussion or, if necessary, by consulting another person in the review team.

Data Charting and Data Synthesis

Data extraction was performed by a single reviewer (EMS/JL/JHP/PC/DCB/KT) and double-checked by a second (EMS/TD). Data charting followed recommendations of the Joanna Briggs Institute [15], with the following data extracted and charted on a standardized form: study characteristics (year of study conduct, country, study design, focus of study), population characteristics, first aid intervention characteristics (e.g., cooling and cooling technique, storage of amputated part, time from injury to any preservation, cold ischemia time), and quantitative outcomes (e.g., number and percent of successful replantations, functional or cosmetic outcomes). The extracted data were synthesized narratively using frequency counting per study type (experimental/animal studies; case report or series; observational studies; systematic reviews) the type/location of injury, the first aid interventions, total ischemic times, percentage of successful replantations, and any delayed (functional/cosmetic) outcomes.

Results

Study Selection

A total of 10,157 records were retrieved from all databases. After the initial screening, 110 full-text records were assessed for eligibility, and 39 were ultimately included. The main reasons for exclusion were the lack of a documented method of preservation/care or the amputated or avulsed part prior to hospital arrival. During the gray literature search, we reviewed 14 first aid guidelines or participant manuals from organizations to identify and compare existing recommendations for the care of completely amputated body parts [16-30]. A list of these organizations can be found in Table 2.

**Table 2:** Organization first aid guidelines and manuals reviewed

Organization
International Federation of Red Cross and Red Crescent [16]
Australia-New Zealand Committee on Resuscitation [17]
St. John Ambulance (U.K) [18]
St. John Ambulance (Canada) [19]
Johanniter International (JOIN) [20]
Singapore Emergency Responder Academy [21]
Singapore Red Cross [22]
African First Aid Materials (AFAM) Project [23]
Emergency Medicine Kenya Foundation/Belgian Red Cross [24]
American Red Cross / American Heart Association [25-27]
National Health Service (U.K.) [28]
Indian Red Cross Society and St. John Ambulance [29]
European Resuscitation Council [30]

**Figure 1:**
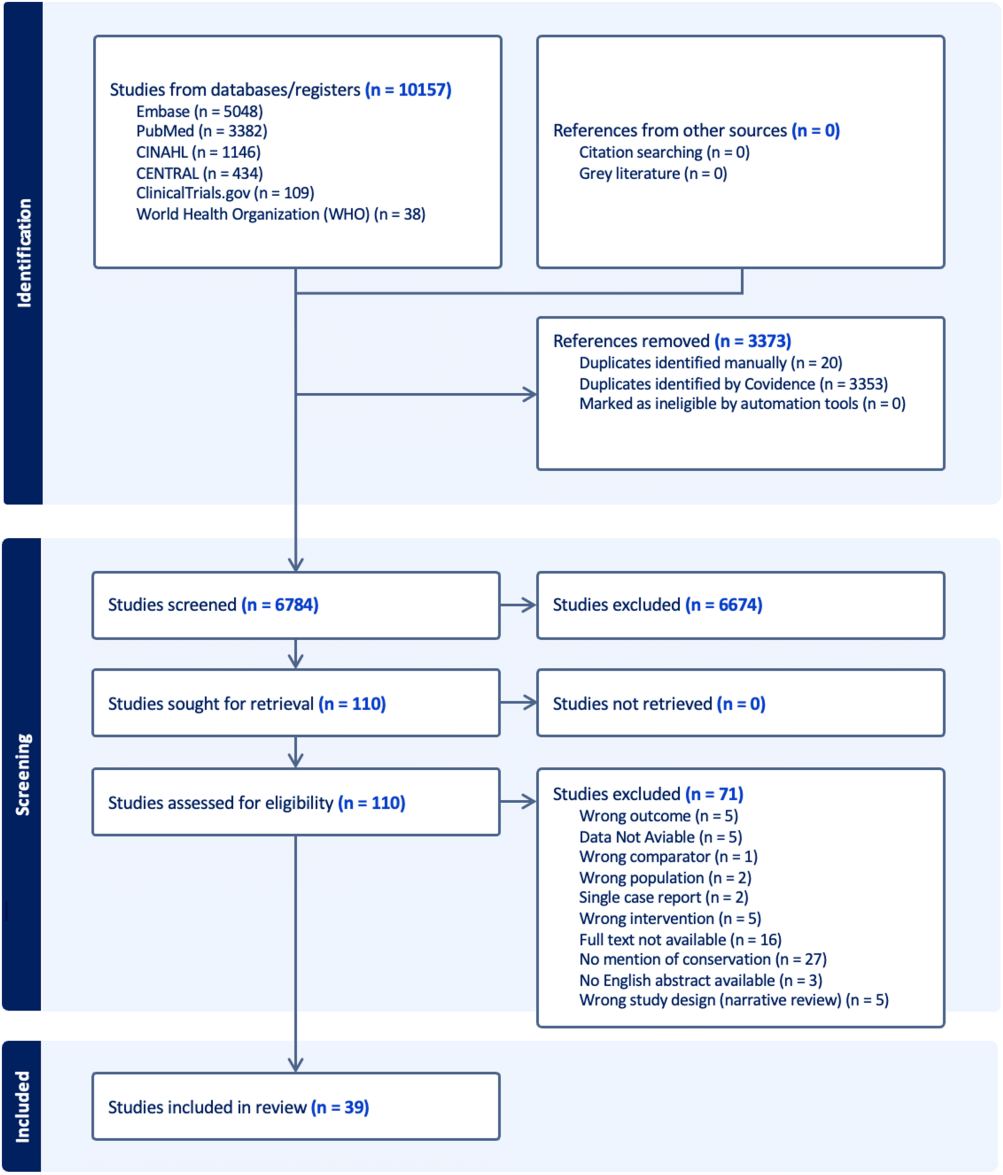
PRISMA flow diagram

The PRISMA flow diagram of the study selection is shown in Figure 1. 

Study Characteristics

Of the 39 records, 24 were case reports [31-54], two were case series [55,56], seven were observational studies [9,11,57-61], two were experimental animal studies [62,63], and four were systematic reviews with meta-analyses [64-67]. A full overview of the characteristics and main findings of the 39 records included is shown in Table 3 and Table 4.

**Table 3:** Study characteristics, case reports, and series.

First author, year, country	Population	Preservation technique	Outcomes reported
Akyurek, 2020, USA [31]	72-year-old female, equestrian accident, scalp avulsion	Avulsed scalp left under snow for four hours before being located	Uncomplicated/complete survival of replanted scalp, normal appearance at four years
Borenstein, 1990, Israel [32]	Two female teenagers with complete avulsion of the scalp and ¾ ears	Case 1: scalp with two auricles wrapped in wet gauze, placed in a plastic bag surrounded by ice, and transferred to the hospital two hours after injury. Case 2: total avulsion of scalp and left auricle. Preservation technique not described.	Case 1: 95% of the scalp and left auricle survived; new hair growth, eyebrow movement at three months post-op. Case 2: partial survival of the scalp, some grafting required; no survival of replanted ears.
Braga-Silva, 2016, Brazil [33]	55-year-old female, amputation of distal ring finger from knife.	Patient presented without amputated part. Amputated finger located later, placed in sealed jar, refrigerated at 4°C for 15 days.	Survival of cold-preserved replanted finger despite 15-day delay. Good function, cosmesis and two-point discrimination at eight-year follow-up.
deLagausie, 2008, France [34]	4 y/o male, amputated penis.	Placed in container with ice without direct contact, 6 hours	Successful replantation of penis after cold ischemic time 6 hours. Normal function at 8 year follow up.
Dvořák, 2020, Czech Republic [35]	38 y/o male, avulsed ear.	Ear wrapped in moistened gauze and stored on dry ice, arrived frozen/rigid.	Successful replantation despite frozen avulsed/amputated ear; Cosmetic changes and cold intolerance on long-term follow up.
El-Bahnasawy, 2024, Saudi Arabia [54]	31 y/o male, penile amputation	Self-inflicted, “placed on ice “by relative, duration of cooling not reported	Replantation at unreported interval after amputation, developed necrosis, subsequent revision; good function and cosmesis on follow up at unreported interval.
Elsahy, 1974, Canada [36]	14 y/o male, avulsed left nasal ala from dog bite.	Tissue lost in a garden for 2 hours. At hospital, was immersed in saline and refrigerated at 7°C for 2 hours before surgery.	Successful grafting 4 hours after injury following 2 hours warm ischemia and 2 hours cold ischemia; Normal skin color at 7 months.
Facio, 2015, Brazil [37]	30 y/o male, amputated penis.	No cooling of amputated part for initial 5 hours, then stored 1 hour in a clean plastic container with saline and ice cubes.	Successful replantation of transplanted penis after 5 hrs warm ischemia; Erectile function, urinary pattern, cosmesis acceptable at 2-yearfollow up.
Fernandez-Palacios, 2009, Spain [38]	28 y/o male, hand amputation at sea.	Hand stored in a plastic bag on ice inside an isothermal box; prolonged transport time due to remote (ocean) location.	Successful replantation at 13 hours post injury; post-transplant infection; recovery at 3 weeks.
Firdaus, 2017, Malaysia [39]	8 y/o male, above elbow amputation from motorcycle accident.	“a witness immediately buys an ice bag from shop nearby and the amputated part was well-preserved.”	Successful replantation of cold-preserved arm. Good circulation in the immediate post-operative period. No further follow up described.
García-Murray, 2009, Mexico [40]	27-year-old female hostage, bilateral ear helix amputations.	Both ears unpreserved for 2 hours, then wrapped in moist gauze, placed in sterile plastic bag, kept in a bucket filled with ice/water and inside a refrigerator 3 hrs pre-op.	Failed replantation after2 hours warm ischemia, 52 hours old ischemia. Successful salvage procedure with reattachment of ears, reconstruction and free flap. Good cosmesis at 12 months.
Gunasagaran, 2022, Malaysia [41]	42 y/o female, left thumb amputation from machete.	Amputated thumb found on side of road (unknown time interval after injury), placed in plastic bag with ice cubes. On arrival 2 hours later, ice cubes had melted, thumb immersed in ice water. Thumb rewashed with normal saline, wrapped with moist gauze, stored in ice box.	Successful replantation despite 2 hours storage directly on ice followed by ice water; No frostbite or maceration of the amputated thumb observed after storage on ice/in ice water. Good motion/function of thumb at follow up.
Henry, 2020, UK [42]	34 y/o male, amputated penis.	No preservation for 15 hours, then put on ice/transported with patient.	Survival of penile transplant despite 15 hours warm ischemia time. Debridement and skin graft needed at 2 months, normal function at 6 weeks.
Kyrmizakis, 2006, Greece [43]	47 y/o male and 20 year/old male, amputated ears.	For both cases, auricles placed in plastic bag with saline, surrounded by ice, transported with patient.	Case 1: successful replantation after 4 hours cold ischemia time as composite graft but required revision at 3 months. No complications at 6 months except 10% decrease in size. Case 2: successful replantation after 3 hours cold ischemia time, composite graft, revision at 3 months.
Li, 2020, China [44]	3 y/o male, right leg amputation at knee level by sword.	No specific care of the amputated leg before hospital arrival (warm ischemia time: 2 hours). Leg then wrapped in saline-soaked gauze, placed in a plastic bag with ice for 400 km transfer to higher level of care. (Cold ischemia time <6 hours).	Successful replantation of leg after 2 hours warm ischemia time, <6 hours cold ischemia. Partial motor and sensory functions 6 months after surgery. During follow-ups, the patient underwent sustained rehabilitation and recovered well.
Liang, 2004, China [45]	30 y/o male, left ear amputation by knife.	Auricle retrieved 5 hours post-amputation. At hospital auricle cleaned and “preserved in ice” for 5 hours.	Successful replantation 10 hours after complete amputation of auricle. Warm ischemia time 5 hours, 5 hours cold ischemia time in-hospital. One year follow up showed color, contour, temperature similar to right ear.
Makki, 2020, Denmark [46]	Case 1: 43 y/o male, amputation of 2/3 upper lip by human bite Case 2: 30 y/o male, ½ upper lip amputated in bicycle-motor vehicle collision.	Avulsed lips both wrapped in saline-soaked gauze and placed on ice in a bag.	At the 8-day follow-up, both patients had 100% healed cleft lip and flap survival. At the 12-month follow-up, case 1 had a cosmetically acceptable result with full movement in the upper lip.
May, 1981, USA [47]	28 y/o male, amputation of 4 fingers of the left hand from a paper cutting machine.	Digits placed in plastic bag surrounded by iced saline. (Unclear if any cooling of digits occurred before arrival at hospital; patient presented with amputated fingers soon after injury.)	Because of the time required to replant all digits, a cold ischemia time of up to 28 hours was recorded for the final digit. All digits survived replantation. The case suggests that the margin of safety in digit replantation may be greater than previously thought.
Musa, 2016, Nigeria [48]	15 y/o male, avulsion of penis from a grinding machine, with scrotal laceration and devitalized tissues.	Initially taken to local hospital and resuscitated. Amputated penis wrapped in gauze at hospital and sent with patient to higher level of care, arrived 30 hours after the injury. No cooling of amputated tissue.	Patient presented 30 hours after injury with the penis mummified precluding reimplantation.
Salem, 2009, Egypt [49]	23 y/o male, penile amputation.	Amputated penis kept dry in plastic bag, double bagged in a contained with ice and slush for 2 hours.	Successful replantation after 2 hours cold ischemia, 5 hours warm (intraoperative) ischemia.
Selmi, 2018, Turkey [50]	11 y/o male, amputation of right testicle from bicycle.	Testicle found in muddy water, cleaned with soap/water, placed in jar of water for 2 hours before seen at emergency department.	No testicular replantation attempted due to storage in water and condition of testicle.
Szlosser, 2015, Poland [51]	82 y/o male, trans- metacarpal amputation of 4 fingers by circular saw.	Amputated fingers "cooled" and "stored appropriately” 3 hours prior to arrival at hospital.	2/4 amputated fingers were replanted, 4 hours warm ischemia (operative) time. At 8 months, minimal movement of fingers; however, because the thumb was uninjured, hand grasp was preserved by the replantation and the patient was satisfied with the result. Author notes that age alone should not be an absolute contraindication to finger replantation.
Usui, 1979, Japan [52]	14 y/o male, left distal 1/3 leg amputation from a mower.	Cooling of the amputated part in ice water; 5-hour transportation time to the hospital.	Successful replantation, 5 hours cold ischemia time. Follow up over 4 years reported no joint contracture or deformity and the child was able to walk and run as fast as other children his age. Success was attributed to the patient’s youth, ideal conditions for nerve repair, and the pre-arrival preservation of the amputated part in ice water.
Wei, 1988, Taiwan [53]	24 y/o female, amputations of right thumb, index and middle fingers and left thumb through small finger by a paper cutting machine.	Prearrival: All 8 digits wrapped in normal saline-soaked gauze and preserved in an ice bag; 76-hour transportation time to the hospital.	All replantations were successful following 76 hour transport time with cold preservation and total cold ischemia times of 84,86 and 94 hours for the left thumb, right thumb and left index finger. At 8 months post-op the patient was able to perform most routine household tasks.
Berger, 1977, Austria [55]	33 patients with 27 complete amputations, 41 incomplete amputations.	Pre-arrival method of preservation or cooling not described except for 4 cases described as “improper first aid contributing to failed replant procedure”, including: - Liquid-filled glass (1) - Floating in ice water (1) - No cooling (2)	Functional replantation not achieved in 9 of 11 cases. Warm ischemia time of more than 8 hours felt responsible for failure of replantation in 2 cases; Review did not clearly describe the specific pre-arrival method of preservation or cooling technique other than for 4 cases. Cold ischemia times of up to 12 hours and a warm ischemia time of up to 6 hours considered the limit for replantation, although consideration of injury mechanism and storage technique were both necessary for exclusion of replantation.
O’Brien, 1973, Australia [56]	8 patients, complete amputation of one or more digits (total 14 amputated digits).	The amputated fingers were "cooled in ice" (n=3), "cooled by ice in a plastic bag" (n=4), and not reported (n=1).	Of 14 digital amputations, 11 survived replantation (83%), with ischemia times of 7 to 14 hours. For preservation methods linked to replantation failures due to complications, one was "cooled in ice," and one was not reported.

**Table 4:** Summary of findings, observational and experimental studies, and systematic reviews

First author, year, country	Study design/population	Preservation techniques
Hayhurst, 1974, Australia [62]	Experimental. 10 Macaque speciosa stump-tailed monkeys; 10 index fingers surgically amputated.	1.5 hours of warm ischemia during amputation. Fingers placed in surgical sponge, moistened with saline, kept at ~4°C for ~21 hours; fingers allowed to return to room temperature for up to 2.5 hours before replantation.
VanGiesen, 1983, USA [62]	Experimental. 40 amputated rabbit ears.	1) Amputated, room air storage, replanted within one hour. 2) Immersed in Ringer's solution at 4°C (24 hours). 3) Not immersed, wrapped in Ringer's moistened sponge at 4°C (24 hours). 4) Immersed in saline at 4°C (24 hours). 5) Not immersed, wrapped in a saline-moistened sponge at 4°C (24 hours). 6) Immersed in Ringer's and wrapped in a dry sponge at room temperature for 24 hours. 7) Frozen, not immersed, wrapped in Ringer's-moistened sponge at 0° to - 5°C for 24 hours. 8) Not immersed, wrapped in Ringer’s-moistened sponge two hours/room air, and 4°C 22 hours.
Li, 2008, China [57]	Observational. 211 patients (117 males and 94 females, mean age 26.2 years (range 1-67)) with 211 complete fingertip amputations undergoing replantation surgery.	1) Dry storage at room temperature (n = 84 digits). 2) Dry storage at 2-6°C (n = 106 digits). 3) Immersed in saline or ethanol (n = 21 digits).
Chen, 2017, China [58]	Observational. 896 amputated fingers (average patient age 22.0±3.8 years).	1) Freeze-dried (n = 536). 2) Room temperature/dry (n = 273). 3) Soaking liquid (n = 87). Specifics of how preservation performed not described.
Okumuş, 2020, Turkey [9]	Observational. 14 patients with amputations of an upper extremity.	All amputated parts but one arrived at the hospital “in properly prepared cold ischemic conditions”. One without cooling had “appropriate” warm ischemic time.
Tark, 1989, Korea [59]	Observational. 261 replantations of amputated digits and hands in 153 patients; 176 were complete amputations.	“Hypothermic” preservation of amputated parts. No description given of timing and method of cooling.
Hoang, 2009, Vietnam [60]	Observational. 10 males with complete forearm amputations.	None of the amputated arms were “properly preserved.”
Sinatro, 2022, USA [11]	Observational. 91 patients with amputations and documented modality of preservation	Prearrival "proper preservation" assessed and defined as "wrapping the part in saline-soaked gauze inside a watertight bag and placing it on ice."
Waikakul, 1998, Thailand [61]	Observational. 186 amputations: palm (24), wrist (75), forearm (50), elbow (9), upper arm (28)	“Good preservation” defined as “cooling” without the description of the technique or time
Huawei, 2015, China [64]	Systematic review. 979 patients with 1755 amputated digits.	Storage in an ice bag or without an ice bag.
Shaterian, 2018, USA [65]	Systematic review. 2 studies with 6,000-digit amputation and replantation cases	“Cold” and “warm” preservation. Details of cooling methods used in the included studies not provided.
Ma, 2016, China [66]	Systematic review. 22 studies, 4,678 amputated digits	Cold (“ice”) preservation; compression bandage (no cooling).
Fijany, 2024, USA [67]	Systematic review: 43 studies, 7344 replanted amputated digits, 1978-2023	1) No cooling. 2) Cooling, methods not described.

Publications originated from 23 different countries in Asia (n = 16), Europe (n = 8), North America (n = 7), South America (n = 2), Australia (n = 2), and Africa (n = 3). Nearly a quarter were published either in the 1970s [36,52,55,56,62] or in the 1980s [47,53,59,63]. All studies included human subjects, except for the two experimental studies that used either a rabbit [63] or monkey [62] animal model.

When looking at equity-relevant data (i.e., participant characteristics that may stratify health opportunities and outcomes) according to the PROGRESS-Plus framework [68,69], most studies only reported on the place (country) of residence for patients, their ethnicity, sex, occupation and age. Other characteristics, such as education and socioeconomic status, were commonly lacking. A summary of equity findings in included studies is shown in Table 5.

**Table 5:** Equity findings

Equity factor	Reported data	Observations
Place of residence	Countries from multiple regions	Highest case reports from Asia (16), Europe (8), and North America (7)
Ethnicity	Not consistently reported	Limited data on ethnic backgrounds
Sex	1669 cases analyzed, 78% male	Higher incidence in males due to occupational hazards
Occupation	Manual laborers and machinery users (45%)	Majority of cases involve workplace injuries
Age	Most affected: 0-5 years (21%), 51-55 years (18%)	Common in children (home injuries) and middle-aged adults (work-related)
Education	Not reported	No direct data on education levels
Socioeconomic status	Implied via occupation and access to care	Workers in hazardous environments at higher risk
Access to healthcare	Variable	Higher replantation success in high-resource settings

Case Reports

Among the 24 case reports, the most frequently amputated/avulsed body parts were fingers (n = 5) [33,41,47,51,53], penis (n = 6) [34,37,42,48,49,54], ear (n = 4) [35,40,43,45], scalp (n = 2) [31,32], and legs (n = 2) [44,52]. Injuries to the nose [36], lips [46], hand [38], arm [39], and testicle [50] were each reported as a single case. Fourteen reports concerned accidental injuries (e.g., accidents with machinery or sharp objects [32,35,38,47,48,51,53], road traffic accidents [39,46,50], horse kick [31], and dog bite [36], whereas nine concerned injuries deliberately inflicted by others [34,37,40,41,43-46,49] (e.g., attack with a sword or knife, human bites). Two case reports covered a self-inflicted penile amputation during a psychotic episode [42, 54].

The three most reported prehospital preservation techniques were (1) wrapping the part in wet/saline-moistened gauze and keeping it cooled (e.g., by placing it in a plastic bag on ice, by placing it directly on dry ice) (n = 5) [32,35,40,46,53,54], (2) immersing the part in cooled liquids (e.g., ice water) (n = 4) [36,37,50,52], and (3) placing the part in a jar or plastic bag and keeping it cooled (e.g., in a refrigerator, inside an isothermal box) (n = 2) [33,38]. In four case reports [34,39,42,51], it was unclear how the part was specifically preserved, but it was clear that it was kept cool during the prehospital period in some way. In four other reports [36,44,45,48], the body parts were brought to the hospital seemingly without any preservation method applied. Ten of the reports described warm ischemia times, ranging from 0 hours [31,39], two hours [36,40,44,50], five hours [37,45], 15 hours [42], to 30 hours [48]. The preservation techniques reported in the included records are shown in Table 6.

**Table 6:** Preservation techniques reported by the included studies

Study	Type of study	Amputated part	Preservation technique	Preservation duration
Hayhurst, 1973, Australia [62]	Animal study	Finger	Wrapped in saline-moistened surgical sponge at 4^o^C	21 hours
VanGiesen, 1983, USA [63]	Animal study	Ear	Immersed in lactated Ringer's solution at 4°C. Wrapped in lactated Ringer's-moistened sponge at 4°C. Immersed in normal saline at 4°C. Wrapped in a normal saline-moistened sponge at 4°C. Immersed in lactated Ringer's at room temperature. Wrapped in lactated Ringer’s-moistened sponge at room temperature. Wrapped in lactated Ringer's-moistened sponge at 0° to - 5°C. Wrapped in lactated Ringer's-moistened sponge at room temperature for two hours and 4°C for 22 hours.	24 hours
Akyurek, 2020, USA [31]	Case report	Scalp	Left under snow	4 hours
Borenstein, 1990, Israel [32]	Case report	Scalp and ear	Wrapped in wet gauze, placed in a plastic bag surrounded by ice	2 hours
Braga-Silva, 2016, Brazil [33]	Case report	Finger	Sealed jar refrigerated at 4^o^C	15 days
deLagausie, 2008, France [34]	Case report	Penis	Placed in container with ice without direct contact	6 hours
Dvořák, 2020, Czech Republic [35]	Case report	Ear	Moistened gauze and stored on dry ice	-
Elsahy, 1974, Canada [36]	Case report	Nose	No preservation for two hours, then immersed in saline, and refrigerated at 7^o^C	2 hours
Facio, 2015, Brazil [37]	Case report	Penis	No preservation for five hours, then stored in a clean plastic container with saline and ice cubes	1 hour
Fernandez-Palacios, 2009 Spain [38]	Case report	Hand	Plastic bag on ice inside an isothermal box	-
Firdaus, 2017, Malaysia [39]	Case report	Elbow	Ice bag	-
García-Murray, 2009, Mexico [40]	Case report	Ear	No preservation for two hours, then wrapped in moist gauze, placed in sterile plastic bag, kept in a bucket filled with ice/water and inside a refrigerator	3 hours
Gunasagaran, 2022, Malaysia [41]	Case report	Finger	No preservation for an unknown duration, then placed in plastic bag with ice cubes, two hours later washed with normal saline, wrapped with moist gauze, and stored in ice box	-
Henry, 2020, UK [42]	Case report	Penis	No preservation, then put on ice	-
Kyrmizakis, 2006, Greece [43]	Case report	Ear	Plastic bag with saline, surrounded by ice	-
Li, 2020, China [44]	Case report	Lower limb	No preservation for two hours, then wrapped in saline-soaked gauze, placed in a plastic bag with ice	-
Liang, 2004, China [45]	Case report	Ear	No preservation for five hours, then cleaned and preserved in ice	5 hours
Makki, 2020, Denmark [46]	Case report	Lip	Wrapped in saline-soaked gauze and placed on ice in a bag	-
May, 1981, USA [47]	Case report	Finger	Plastic bag surrounded by iced saline	-
Musa, 2016, Nigeria [48]	Case report	Penis	Wrapped in gauze	-
Salem, 2009, Egypt [49]	Case report	Penis	Kept dry in plastic bag, double bagged in a container with ice and slush	2 hours
Selmi, 2018, Turkey [50]	Case report	Testicle	Placed in a jar of water	2 hours
Szlosser, 2015, Poland [51]	Case report	Finger	Cooled and stored without further details	3 hours
Usui, 1979, Japan [52]	Case report	Lower limb	Cooling in ice water	5 hours
Wei, 1988, Taiwan [53]	Case report	Finger	Wrapped in normal saline-soaked gauze and preserved in an ice bag	76 hours
Berger, 1977, Austria [55]	Case series	Not mentioned	Described in four cases: liquid-filled glass (1), floating in ice water (1), no cooling (2)	-
O’Brien, 1973, Australia [56]	Case series	Finger	Described in seven cases: cooled in ice (3), cooled by ice in a plastic bag (4)	-
Li, 2008, China [57]	Retrospective observational study	Finger	Dry storage at room temperature, dry storage at 2-6^o^C, immersed in saline or ethanol	-
Chen, 2017, China [58]	Retrospective observational study	Finger	Freeze-dried, room temperature, soaked in liquid but not described	-
Okumuş, 2020, Turkey [9]	Retrospective observational study	Upper limb	Cold condition but not described	-
Tark, 1989, Korea [59]	Retrospective observational study	Finger and hand	Hypothermic preservation but not described	-
Hoang, 2009, Vietnam [60]	Retrospective observational study	Upper limb	Not described/no hypothermic preservation	-
Sinatro, 2022, USA [11]	Retrospective observational study	Not mentioned	Wrapped in saline soaked gauze inside a watertight bag and placing it on ice vs other methods but not described	-
Waikakul, 1998, Thailand [61]	Prospective observational study	Upper limb	Cooling but not described	-
Huawei, 2015, China [64]	Systematic review and meta-analysis	Finger	Storage in an ice bag, No storage in an ice bag but not described	-
Shaterian, 2018, USA [65]	Systematic review and meta-analysis	Digit	Cold but not described, warm or room temperature but not described	-
Ma, 2016, China [66]	Systematic review and meta-analysis	Digit	Cold but not described, compression bandage	-

Case Series

In their case series of 33 patients with 68 amputated parts, Berger et al. [55] did not describe the specific tissues that were amputated and did not specify the pre-arrival method of care or preservation of amputated parts, except for four cases that were reported to have “improper first aid contributing to failed replantation procedure”: lack of cooling in two cases, placing the amputated parts in a liquid-filled glass, and placing the part in ice water.

The second case series [56] of eight patients with 14 amputated digits reported that except for one case, all amputated digits were cooled as soon as possible after injury by placing them in a plastic bag and then inserting the digit(s) into another bag containing ice.

Observational Studies

A total of 1,669 patients with amputations were included in the seven observational studies that had sample sizes ranging from 10 [60] to 896 [58] patients per study.

The most frequently studied amputated body parts were finger(tip)s [57,58], fingers and hands [59], upper extremities [9,60,61], and upper and lower extremities [11]. Reported preservation methods included (1) dry storage at room temperature [57,58], (2) dry storage at 2-6°C [57], (3) immersion in liquids (e.g., saline, ethanol) [57,58], (4) freeze-drying [58], and (5) wrapping the part in saline-soaked gauze inside a watertight bag and placing it on ice [11]. Four of the studies did not specify the method but only referred to “properly prepared cold ischemic conditions” [9], “hypothermic preservation” [59], or “proper preservation” [60] or described cooling as “good preservation” without further description [61].

Experimental Animal Studies

Two studies assessed the survival of replanted amputated parts using experimental animal models. In Hayhurst [62], 10 amputated index fingers of Macaque monkeys were wrapped in saline-moistened gauze sponge and maintained at 4°C for 21 hours, then brought to room temperature for up to 2.5 hours before replantation.

VanGiesen [63] compared different preservation methods for amputated ears of rabbits, including 24-hour storage at room temperature, at 4°C or at -5°C, with the part either wrapped in saline or lactated Ringer’s solution-moistened gauze/sponge or immersed in these liquids.

Systematic Reviews

In three systematic reviews [64,66,67], the association between non-surgical factors and survival rates of digital replantation were examined. In the first review [64], studies were included if they reported on single or multiple amputated finger replantation patients who were followed up for at least six months and if they had extractable replantation survival data. It included two master's theses [70,71] and two studies [72,73] investigating the relationship between the method of preservation (preserved in an ice bag vs. preserved at a common temperature) and survival rates in 929 participants. The second review [66] applied similar selection criteria but excluded case series with less than 10 patients and studies reporting only successful replantations. It included three unreferenced studies examining the association between the method of preservation (cold preservation vs. emergency compression bandage) and survival rates. The third review [67] evaluated the association of both temperature and total ischemia time on replantation success, which was defined as fingertip necrosis of <25% of the replant. The review included 43 studies with 7,344 replanted digits. Case series of ≤15 patients were excluded. However, the review does not distinguish between pre-arrival times for cold preservation vs in-hospital cold storage times, nor are methods of cold preservation described. A fourth review [65] aimed to identify patient- and surgery-related factors influencing replantation survival of amputated fingers. Studies were only included if they provided data for complete amputations. This review included two studies [57,72] that allowed investigating the effect of the method of preservation.

Guidelines From Organizations That Produce First Aid Manuals or Guidelines

Of the 13 international organizations identified with published first aid manuals or guidelines (Table 2), all but two [17,29] provided specific guidance for management, including cold storage of completely amputated/avulsed body parts. The remaining guidelines and manuals all emphasize three critical actions in managing amputations: rapid hemorrhage control, proper (cold) preservation of the severed part, and urgent transport to a medical facility. Key recommendations include care of the injured person by applying direct pressure to control bleeding and, if not controlled, to apply a tourniquet; securing the amputated part in a sterile, cool environment without direct contact with ice; and ensuring prompt Emergency Medical Services activation. Most guidelines advise against washing the severed part, wrapping the part in sterile gauze or a clean cloth moistened with saline, followed by placing the part in a watertight plastic bag or wrapped in cling film. The bag is then placed in a second container with ice or chilled water, ensuring the amputated part does not come into direct contact with ice, labeled with time of injury/name, and transported with the injured person, if possible. There is little variation in guideline recommendations.

Results of individual studies

Replantation Attempt Rates

In one observational study [11], replantation was attempted at a significantly lower rate in patients with “improperly preserved” amputated parts compared to those parts wrapped in saline-soaked gauze inside a watertight bag on ice.

Survival of Replanted Amputated Parts

Survival of replantation was the most frequently assessed outcome; only one of the observational studies did not report it [11]. For the individual findings of the studies or publications, the reader is referred to Tables 3-4. In the case reports, all attempted replantations were successful despite the variations in amputated parts and preservation techniques and despite warm ischemia times running up to 30 hours. Similarly, the two case series [55,56] reported on replantation survival after up to 14 hours of warm ischemia. Both case series linked several replantation failures to improper first aid or “cooling in ice."

The findings of the observational studies confirm that the preservation method is associated with the survival of the amputated part. Dry cool storage at 2-6°C was shown to be associated with higher survival than immersion in saline or ethanol [57]. In another study, the process of freeze-drying was associated with higher survival than dry storage at room temperature and immersion in liquid [58]. Two studies showed that cooling was better than no cooling [9,59]. One study reported that survival of replanted arms and hands can be successful despite a lack of any first aid cooling and a delay of up to 13 hours between injury and hospital arrival, although functional outcomes were rated as fair to poor in six out of 10 cases [60].

The two experimental animal studies [62,63] confirm that cooling to 4°C is the preferred method of preservation and that freezing and storing amputated parts at room temperature leads to failure of replantation.

Although the results of the meta-analyses performed in the four identified systematic reviews should be considered with caution, as they applied inconsistent eligibility criteria and displayed methodological flaws (e.g., lack of transparent reporting), their findings also suggest that replantation of amputated digits that are stored cool are more likely to survive than those stored at common temperature [64,65,67] or using compression bandaging [66].

Functional Outcomes

Three observational studies reporting on functional outcomes indicate that cold preservation might have a substantial beneficial effect. In the study by Okumuş et al. [9], for patients whose amputated arms and hands arrived at hospital “in properly prepared cold ischemic conditions” all but one were all satisfied with the recovery of mobility, active motion of digits and active movement of joints, recovery of sensitivity, and the ability to perform daily works with their replanted limbs. Similarly, in the study by Waikakul et al. [61], all patients were satisfied with their replanted limbs. “Good preservation of the amputated segment”, which consisted of cooling, had a significant beneficial effect on the functional outcome. In contrast, in the observational study by Hoang et al. [60], in which no cooling efforts were undertaken for any of 10 amputated arms, functional outcomes were rated as fair to poor in 60% of patients.

Discussion

The current scoping review provides a comprehensive overview of the evidence on the initial preservation of amputated and avulsed body parts. In total, we identified 34 case reports, series, and observational studies and four systematic reviews from 24 countries worldwide, published as far back as the 1970s.

Success of Replantation

The bulk of the evidence related to prearrival storage of amputated parts was provided by 24 case reports and two case series that described various methods and durations of hypothermic storage of amputated body parts. Despite differences in body parts amputated and ways of providing cold storage, replantation was deemed successful in nearly all cases, with acceptable cosmesis and functional outcomes on follow-up. However, we should be aware of a possibly strong publication bias, as case reports generally describe treatment successes rather than failures [74, 75]. In addition, it is important to recognize that survival of a replanted amputated part does not equate to “success”, the latter of which may relate to long-term functional, sensory, cosmetic, and patient satisfaction outcomes. In several case reports, the reporting of follow-up outcomes was limited or not included.

Pre-arrival Hypothermic Preservation

Most of the observational studies and two out of four systematic reviews reported an association between pre-arrival hypothermic preservation and successful replantation, including acceptable function and cosmesis on follow up [9,57,59,61], while one systematic review [67] found greater survival of replanted digits stored cold for 12 or more hours compared with no cold storage. Replanted amputated digits and avulsed ear/nasal tissue (which lack skeletal muscle) appear to tolerate a lack of hypothermic preservation for longer periods than more proximal extremity amputations. However, one observational study [60] reported successful replantation of 10/10 amputated arms despite a lack of pre-arrival hypothermic preservation and a 2- to 8-hour delay between injury and hospital arrival; functional outcomes were rated as fair to poor for 6/10 patients. The author notes that in some cases, from a psychological and social perspective, a person with an amputated extremity would prefer to have the extremity replanted even if it leads to a limited functional outcome, rather than to have an extremity with a stump or prosthesis.

Techniques for Hypothermic Preservation

Early experimental studies with animal models showed improved survival of replanted amputated parts following cooling of the part to 4°C for up to 24 hours [62,63]. These studies likely led to current recommendations in the orthopedic literature describing "proper" prehospital preservation techniques using cold but nonfreezing storage, such as by wrapping the part in saline moistened gauze to avoid direct contact with ice, placing the wrapped part in a watertight bag and then placing the bag inside another container holding ice or an ice-water mixture [7].

Implications for First Aid and Prehospital Care

One observational study [11] has implications for first aid and Emergency Medical Services education. Sinatro et al. [11] reported that in nearly two-thirds of patients with amputations, proper preservation (i.e., hypothermic storage) was not used before hospital arrival and led to a significantly lower rate of attempted replantation compared with properly preserved parts. Although this is one study from a single country, several of the included case reports either did not use cold preservation techniques or were delayed for two to five hours. In some cases, this was due to the amputated part being lost or intentionally withheld, but in other cases, it may reflect a lack of knowledge of proper cold preservation techniques or a lack of resources. For studies that reported details on how cold preservation was achieved before hospital arrival, we observed a wide variety of means to create a cold storage temperature, suggesting that the “proper” method of double bagging on ice or an ice-water mixture described in the orthopedic literature may be modifiable for the setting and circumstances. One case in which an avulsed scalp was lost in the snow before being retrieved described the tissue as being pliable and without apparent freezing damage [31].

Equity Considerations

The findings from this scoping review highlight concerns about first aid practices in regions lacking resources for the optimal cooling of amputated body parts. Essential items such as ice, watertight plastic bags, and suitably sized containers are not universally accessible. While some case reports document successful replantation of body parts without prior cold preservation, there is a pressing need for studies that evaluate various cooling methods and temperature conditions. For instance, comparing techniques like placing an amputated part in a watertight bag stored in an isothermal cooler with instant cold packs, using a battery-powered cooler, or immersing the part in a water bath cooler than ambient temperature would be valuable. Conducting such studies, along with detailed reporting on equity-related factors, could help mitigate existing health disparities.

Limitations

The strength of the current scoping review includes its rigorous and systematic methodology. A major limitation of this scoping review was the difficulty identifying the method of cooling an amputated part, particularly in the observational studies and systematic reviews. In some studies, preservation was simply described as “cold vs warm” or as “proper preservation." In addition, it was not always clear how soon cooling was initiated after injury or how it was maintained for long transport times. We, therefore, recommend that future studies should provide greater detail related to cooling methods, maintenance of cooling, time between amputation and initiation of cooling, and time to hospital arrival.

## Conclusions

This scoping review identified an extensive evidence base on the initial preservation of amputated and avulsed body parts. The studies indicate that nonfreezing cold preservation of the parts as soon as possible after injury is associated with survival of replantation, improved function and cosmesis at follow-up, and allows amputated tissue to tolerate a longer ischemic time before replantation. Additional studies are needed to identify alternative means of providing sustained cold storage of amputated body parts in resource-limited first aid settings worldwide to help reduce health inequities.

## Appendices

Appendix A

**Table 7:** Preferred Reporting Items for Systematic reviews and Meta-Analyses extension for Scoping Reviews (PRISMA-ScR) Checklist

Section	Item	PRISMA-ScR checklist item	Reported on page no.
Title	1	Identify the report as a scoping review.	1, Lines 1-2
ABSTRACT			
Structured summary	2	Provide a structured summary that includes (as applicable): background, objectives, eligibility criteria, sources of evidence, charting methods, results, and conclusions that relate to the review questions and objectives.	1, Lines 6-28
INTRODUCTION			
Rationale	3	Describe the rationale for the review in the context of what is already known. Explain why the review questions/objectives lend themselves to a scoping review approach.	3, Lines 33-63
Objectives	4	Provide an explicit statement of the questions and objectives being addressed with reference to their key elements (e.g., population or participants, concepts, and context) or other relevant key elements used to conceptualize the review questions and/or objectives.	3, 65-75
Methods			
Protocol and registration	5	Indicate whether a review protocol exists; state if and where it can be accessed (e.g., a Web address); and if available, provide registration information, including the registration number.	NA
Eligibility criteria	6	Specify characteristics of the sources of evidence used as eligibility criteria (e.g., years considered, language, and publication status), and provide a rationale.	3-4, Lines 88-90
Information sources*	7	Describe all information sources in the search (e.g., databases with dates of coverage and contact with authors to identify additional sources), as well as the date the most recent search was executed.	4, Lines 92-104
Search	8	Present the full electronic search strategy for at least 1 database, including any limits used, such that it could be repeated.	Supplementary material
Selection of sources of evidence†	9	State the process for selecting sources of evidence (i.e., screening and eligibility) included in the scoping review.	4, Lines 106-109
Data charting process‡	10	Describe the methods of charting data from the included sources of evidence (e.g., calibrated forms or forms that have been tested by the team before their use, and whether data charting was done independently or in duplicate) and any processes for obtaining and confirming data from investigators.	4, Lines 111-122
Data items	11	List and define all variables for which data were sought and any assumptions and simplifications made.	5, Lines 121-127.
Critical appraisal of individual sources of evidence§	12	If done, provide a rationale for conducting a critical appraisal of included sources of evidence; describe the methods used and how this information was used in any data synthesis (if appropriate).	NA
Synthesis of results	13	Describe the methods of handling and summarizing the data that were charted.	6, Lines 129-146
Results			
Selection of sources of evidence	14	Give numbers of sources of evidence screened, assessed for eligibility, and included in the review, with reasons for exclusions at each stage, ideally using a flow diagram.	Figure 1
Characteristics of sources of evidence	15	For each source of evidence, present characteristics for which data were charted and provide the citations.	Figure 1
Critical appraisal within sources of evidence	16	If done, present data on critical appraisal of included sources of evidence (see item 12).	NA
Results of individual sources of evidence	17	For each included source of evidence, present the relevant data that were charted that relate to the review questions and objectives.	Tables 2-4
Synthesis of results	18	Summarize and/or present the charting results as they relate to the review questions and objectives.	5-10, Lines 124-288
Discussion			
Summary of evidence	19	Summarize the main results (including an overview of concepts, themes, and types of evidence available), link to the review questions and objectives, and consider the relevance to key groups.	10, Lines 285-343
Limitations	20	Discuss the limitations of the scoping review process.	12, Lines 345-353
Conclusions	21	Provide a general interpretation of the results with respect to the review questions and objectives, as well as potential implications and/or next steps.	13, Lines 355-362
Funding			
Funding	22	Describe sources of funding for the included sources of evidence, as well as sources of funding for the scoping review. Describe the role of the funders of the scoping review.	Page 13, Lines 365-367

Appendix B

**Table 8:** ILCOR scoping review plan

PICOST	Description (with recommended text)
Population	Adults and children with a traumatic complete amputation or avulsion of a body part (e.g. digit, hand, arm) or soft tissue in the out-of-hospital setting
Intervention	Any approach to preservation of the amputated body part or avulsed tissue for possible replantation/attachment
Comparison	Another approach to preservation of the amputated body part or avulsed tissue for possible replantation/attachment
Outcomes	Any clinical outcome. (preset text)
Study Design	Randomized controlled trials (RCTs) and non-randomized studies (non-randomized controlled trials, interrupted time series, controlled before-and-after studies, cohort studies) are eligible for inclusion. Grey literature and social media and non-peer reviewed studies, unpublished studies, conference abstracts and trial protocols are eligible for inclusion. If it is anticipated that there will be insufficient studies from which to draw a conclusion, case series may be included in the initial search. The minimum number of cases for a case series to be included can be set by the lead author after discussion with the priority team or task force. All relevant publications in any language are included as long as there is an English abstract.
Time frame	All years
Definitions	*Traumatic amputation*: an injury to an extremity that results in the immediate loss or removal of an extremity or digit(s). Traumatic amputations occur accidentally, such as in an occupational or home setting, or the result of battlefield conflict, such as due to an explosion. In a partial amputation, some tissue remains intact, while a complete amputation separates the limb or digit from the body, usually including bone, soft tissue, accompanying blood vessels nerves, muscle or tendon. Avulsion: the traumatic tearing away or forcible separation of skin and underlying soft tissue from the body, leading to exposure of underlying tendons, muscle and connective tissue. A complete avulsion results in the separation of the avulsed skin and tissue away from the body, such as an ear or other soft tissue that is caught in a sharp object, while in a partial avulsion there is an attached base or stalk. Replantation: a restorative surgery for the reestablishment of the anatomic integrity of major structures of an extremity in complete or partial amputation to regain the viability of the extremity and attain the acceptable functional outcome.
Background and Rationale for this PICOST	Complete amputation of extremities or digits is a physically and emotionally traumatic experience that can lead to long-term disability. Globally, the incidence and prevalence number of traumatic amputation increased from 11.37 million and 370.25 million in 1990, to 13.23 million and 552.45 million in 2019, with a raise of 16.4 and 49.2%, respectively [1]. Causes of amputations in the U.S. are motor vehicle collisions (51%), industrial accidents (19%) and agricultural accidents (10%) [2]. Data from the US National Electronic Injury Surveillance System database [2] between 2012 and 2021 identified 7323 patients with traumatic amputation, with the most frequent age group being 0-5 years age, followed by 51-55 years. Fingers were most frequently amputated (91%), followed by toes (5%). In this database, most injuries occurred in the home (56%) and were due to doors, bench or table saws, and power lawn mowers. In some case and in countries with higher resources, when the amputated extremity or digit is recovered and transported with the patient, the digit or limb can be successfully replanted. The proper storage of an ischemic amputated limb or digit is essential to improve the potential for replantation surgery. Indications for replantation have increased over the past few decades. When replantation is not an option, skin from the amputated limb can sometimes be used for grafting back over the defect created by the avulsion or amputation. A complete avulsion can be a disfiguring wound that is difficult to repair. Properly preserve avulsed tissue can similarly be used as a biologic wound cover, and skin harvested for grafting. Cooling completely avulsed soft tissue or an amputated body part while protecting the part from freezing is considered the gold standard for preserving ischemic tissue and improving the chances of a successful replantation or reattachment. First Aid guidelines suggest wrapping the amputated/avulsed body part in saline-moistened gauze, placing the body part/tissue in a plastic bag or airtight container, and placing the bag or container in a second bag /container of ice and water. However, it has been reported that only 35% of patients with traumatic amputations present to the emergency department with properly preserved amputated body parts, making it difficult for surgeons to offer replantation when it would be an option. This PICOST was prioritized based on the global burden of traumatic amputations, awareness of ILCOR Task Force members of issues with improper preservation of amputated/avulsed body parts, and the subsequent potential lost opportunity for replantation or reattachment of amputated or avulsed body parts. First aid for the patient’s wound is not included in this review as it has been covered in other ILCOR reviews (FA530, Control of life-threatening bleeding with direct pressure; FA 1543 and 1549, Tourniquets for life-threatening bleeding from an extremity; FA 7334, Use of hemostatic dressings for life-threatening bleeding).
Task Force Suggested Outcomes	All clinical outcomes, including successful replantation/reattachment of amputated/avulsed body parts, cosmesis and functional outcomes will be included in the scoping review search. If no prior search, suggested specific search terms/keywords: Amputation; avulsion; trauma; replantation; preservation.
Suggested specific search terms/keywords	amputation; avulsion; trauma; replantation; preservation.

Appendix C

**Table 9:** Search strategies

PubMed
1: (“Amputation, Traumatic”[MeSH] OR ((amput*[TIAB] OR avulsion*[TIAB] OR avulsed[TIAB] OR severed[TIAB] OR transected[TIAB]) AND (complete[TIAB] OR completely[TIAB] OR total[TIAB] OR totally[TIAB] OR entire[TIAB] OR entirely[TIAB] OR entirety[TIAB] OR trauma[TIAB] OR traumatic[TIAB] OR accident*[TIAB] OR injur*[TIAB]))) NOT (“Amputation, Surgical”[MeSH] OR “Tooth Avulsion”[MeSH] OR dental[TIAB] OR tooth[TIAB] OR teeth[TIAB])
2. “Tissue preservation”[MeSH] OR preserv*[TIAB] OR conserv*[TIAB] OR cooling[TIAB] OR cooled[TIAB] OR ice[TIAB] OR wrapping[TIAB] OR wrapped[TIAB] OR wrap[TIAB] OR moist*[TIAB] OR damp*[TIAB] OR cleanse[TIAB] OR cleansed[TIAB] OR cleansing[TIAB] OR storing[TIAB] OR storage[TIAB] OR stored[TIAB] OR immers*[TIAB]
3: 1 AND 2
Embase
('Traumatic amputation'/exp OR ((amput*:ab,ti OR avulsion*:ab,ti OR avulsed:ab,ti OR severed:ab,ti OR transected:ab,ti) AND (complete:ab,ti OR completely:ab,ti OR total:ab,ti OR totally:ab,ti OR entire:ab,ti OR entirely:ab,ti OR entirety:ab,ti OR trauma:ab,ti OR traumatic:ab,ti OR accident*:ab,ti OR injur*:ab,ti))) NOT (dental:ab,ti OR tooth:ab,ti OR teeth:ab,ti)
'Tissue preservation'/exp OR preserv*:ab,ti OR conserv*:ab,ti OR cooling:ab,ti OR cooled:ab,ti OR ice:ab,ti OR wrapping:ab,ti OR wrapped:ab,ti OR wrap:ab,ti OR moist*:ab,ti OR damp*:ab,ti OR cleanse:ab,ti OR cleansed:ab,ti OR cleansing:ab,ti OR storing:ab,ti OR storage:ab,ti OR stored:ab,ti OR immers*:ab,ti
1 AND 2
CENTRAL
([mh “Amputation, Traumatic”] OR amput*:ti,ab,kw OR avulsion*:ti,ab,kw OR avulsed:ti,ab,kw OR severed:ti,ab,kw OR transected:ti,ab,kw) NOT ([mh “Tooth Avulsion”] OR dental:ti,ab,kw OR tooth:ti,ab,kw OR teeth:ti,ab,kw)
[mh “Tissue preservation”] OR preserv*:ti,ab,kw OR conserv*:ti,ab,kw OR cooling:ti,ab,kw OR cooled:ti,ab,kw OR ice:ti,ab,kw OR wrapping:ti,ab,kw OR wrapped:ti,ab,kw OR wrap:ti,ab,kw OR moist*:ti,ab,kw OR damp*:ti,ab,kw OR cleanse:ti,ab,kw OR cleansed:ti,ab,kw OR cleansing:ti,ab,kw OR storing:ti,ab,kw OR storage:ti,ab,kw OR stored:ti,ab,kw OR immers*:ti,ab,kw
1-2
CINAHL
((MH "Amputation+") OR TI "amput*" OR AB "amput*" OR TI "avulsion*" OR AB "avulsion*" OR TI avulsed OR AB avulsed OR TI severed OR AB severed OR TI transected OR AB transected) NOT ((MH “Tooth Injuries+”) OR TI “dental” OR AB “dental” OR TI “tooth” OR AB “tooth” OR TI “teeth” OR AB “teeth”)
(MH "Tissue Preservation+") OR TI "preserv*" OR AB "preserv*" OR TI "conserv*" OR AB "conserv*" OR TI cooling OR AB cooling OR TI cooled OR AB cooled OR TI ice OR AB ice OR TI wrapping OR AB wrapping OR TI wrapped OR AB wrapped OR TI wrap OR AB wrap OR TI "moist*" OR AB "moist*" OR TI "damp*" OR AB "damp*" OR TI cleanse OR AB cleanse OR TI cleansed OR AB cleansed OR TI cleansing OR AB cleansing OR TI storing OR AB storing OR TI storage OR AB storage OR TI stored OR AB stored OR TI “immers*” OR AB “immers*”
1-2
3 AND Journal Subset limited to ‘Peer reviewed’ AND Publication Date
ClinicalTrials.Gov
Condition/disease: amputation OR amputated OR avulsion OR avulsed OR severed OR transected
Intervention/treatment: preservation OR preserved OR preserve OR conservation OR conserved OR conserve OR cooling OR cooled OR ice OR wrapping OR wrapped OR wrap OR moist OR damp OR cleanse OR cleansed OR cleansing OR storing OR storage OR stored OR immersed OR immersing
WHO ICTRP
Advanced search: trials with the exact phrase or contains:
Amputat* OR avuls* OR severed OR transected in the Condition
Preserv* OR conserv* OR cool* OR ice OR wrap* OR moist* OR damp* OR cleans* OR stor* OR immers* in the Intervention
